# Myelodysplastic syndrome in a case of new‐onset pancytopenia

**DOI:** 10.1002/ccr3.5533

**Published:** 2022-03-03

**Authors:** Ian Lancaster, Deep Patel, Vikas Sethi, Weston Connelly, Joseph Namey

**Affiliations:** ^1^ HCA Healthcare/USF Morsani College of Medicine GME Programs Largo Medical Center Largo Florida USA

**Keywords:** myelodysplastic syndrome, pancytopenia, piperacillin/tazobactam‐induced immune response, vaccine‐associated pancytopenia

## Abstract

Myelodysplastic syndrome (MDS) is an infrequent cause of pancytopenia, which is a decrease in all three peripheral blood cell lines. We report the case of new‐onset pancytopenia following administration of a COVID‐19 vaccine and recurrent Zosyn use who was later found to have myelodysplastic syndrome.

## Abstract 2

Myelodysplastic syndrome (MDS) is a group of heterogeneous clonal hematopoietic stem cell malignancies. More than 100 genes have been found to be recurrently mutated in myelodysplastic syndrome, and these encode components of the spliceosome, chromatin remodeling factors, epigenetic pattern modulators, and transcription factors.

## 1 INTRODUCTION

Pancytopenia is a hematologic condition characterized by a decrease in all three peripheral blood cell lines, resulting in anemia, thrombocytopenia, and leukopenia.^1^, ^2^ The signs and symptoms of pancytopenia are variable and dependent on the cell line or lines that are affected. Common signs include malaise, fatigue, easy bruising, and recurrent nosebleeds.^3^, ^4^ Life‐threatening complications include hemodynamic instability, hypoxia, febrile neutropenia and sepsis, and hemorrhage.^3^, ^4^ There are a myriad of causes and etiologies for pancytopenia which can be broken down into three categories: (1) impaired production, (2) peripheral destruction, and (3) impaired production and peripheral destruction.^3^, ^4^

We report the case of an individual with new‐onset pancytopenia which had been found to be due to a case of myelodysplastic syndrome (MDS) on bone marrow biopsy at an outside healthcare facility. Unique in this patient's case, he was administered his first dose of an mRNA COVID‐19 vaccine approximately one week prior to hospital admission and one day prior to symptom onset. His case raised the question of whether the COVID‐19 mRNA vaccine may have been responsible or associated with his pancytopenia. In this case, we review and discuss the evaluation of pancytopenia as well as the clinical presentation of MDS.

## 2 CASE

A 64‐year‐old Caucasian male patient with a past medical history of chronic obstructive pulmonary disease (COPD) presented to the emergency department for 6 days of worsening abdominal pain and fever of 102F which occurred one day following the administration of his first dose of an mRNA COVID‐19 vaccine in March 2021. Prior to hospitalization, he was recommended to undergo a screening colonoscopy following a positive fecal occult blood test. His laboratory work in the ED was significant for a white blood cell cout of 7.6 × 10^9^/L, platelet count of 147 × 10^9^/L, and a hemoglobin of 10.4 g/dl with a mean corpuscular volume of 104.1 fL. His complete metabolic panel was ordered and grossly unremarkable and included a BUN 16 mg/dL and creatinine 1.19 mg/dL. His lipase was 47 U/L. Urine, blood, and stool cultures were obtained on presentation. Computed tomography (CT) scan of the abdomen/pelvis demonstrated a narrowing of the colon at the area of the splenic flexure concerning for a neoplastic or infectious process. The patient was treated empirically with intravenous piperacillin/tazobactam in addition to supportive care with intravenous pain control and intravenous fluids. He was then admitted to the medical/surgical floor, and gastroenterology was consulted with a plan for colonoscopy for further evaluation of the area of narrowing identified on CT.

The hospital day two, the patient developed pancytopenia with a white blood cell count of 3.7 x 10^9^/L, thrombocytopenia with a platelet count of 74 x 10^9^/L, and persistent macrocytic anemia with a hemoglobin of 8.2 g/dl. White blood cell differential at that time was significant for segmented neutrophil 13%, band neutrophil 19%, lymphocytes 52%, monocytes 1%, and atypical lymphocytes 3%. He was not given heparin prior to these laboratory results. Later in the day, the colonoscopy was canceled as the patient became hypotensive and a lactic acidosis of 4.49 mmol/L. He was transferred to the intensive care unit.

In the intensive care unit, emphasis was placed on maintaining hemodynamic stability and the patient received multiple fluid boluses with subsequent improvement in his blood pressure. In addition, he also received stress dose steroids with intravenous methylprednisolone. Concurrently, investigation of the pancytopenia began, and hematology/oncology was consulted. Notable laboratory work on the day following transfer to intensive care unit showed macrocytic anemia with a hemoglobin of 8.2 g/dl and mean corpuscular volume of 104.1 fL, an absolute reticulocyte count of 0.4 10^9^/L, an elevated erythrocyte sedimentation rate (ESR) of 124 mm/h, an elevated haptoglobin of 446 mg/dl, and peripheral smear demonstrating macrocytosis, monocytosis, and few immature myeloid cells. Iron panel demonstrated an elevated ferritin of 970 mcg/L, iron of 29 µg/dl, and total iron‐binding capacity of 202 µg/dl. Vitamin B12 and folate levels were within normal limits. Coagulation studies demonstrated an elevated partial thromboplastin time (PTT) of 37 s, elevated fibrinogen of 860 mg/dl, and an elevated D‐dimer of 6.54 mcg/mL with a normal international normalized ratio (INR) of 1.17 and prothrombin time (PT) of 12.4 s. His blood, urine, and stool cultures all remained unremarkable. It was suspected that the patient's pancytopenia was likely multifactorial in nature with low suspicion for marrow infiltrative causes given the acute nature of the pancytopenia and no medications were discontinued.

Next, as the patient's hemodynamic stability improved, he was then transferred out of the intensive care unit on hospital day four. A colonoscopy was planned and later performed on hospital day five for evaluation of the narrowing of the splenic flexure identified on intra‐abdominal imaging. Leading up to the colonoscopy the patient's anemia and leukopenia remained unchanged, but his thrombocytopenia continued to worsen and reached a nadir platelet count of 37 × 10^9^/L. This necessitated the administration of two units of platelets prior to the procedure.

Endoscopy revealed two polyps, one 5 mm polyp in the descending colon and a 10 mm pedunculated polyp approximately 15 cm from the anal verge. Due to the findings on colonoscopy and lack of an infectious etiology, hematology recommended a bone marrow biopsy prior to hospital discharge. Unfortunately on hospital day six, the patient left against medical advice prior to the biopsy, but was given instruction to follow‐up as an outpatient with hematology and gastroenterology. As the patient was afebrile and hemodynamically stable, he did not receive additional treatments for his pancytopenia. At the time of discharge, his CBC had shown a white blood cell count 2.5 × 10^9^/L, hemoglobin of 7.5 g/dl, and platelet count of 78 × 10^9^/L. The differential at that time had included neutrophils 38%, lymphocytes 40%, and monocytes 21%. Following discharge, the biopsies from colonoscopy demonstrated a tubular adenoma in the descending colon and tubule‐villous adenoma of the sigmoid colon.

The etiology of this patient's pancytopenia was unclear at the time of hospital discharge, and a wide differential diagnosis was considered including hematologic neoplasms, response to an intra‐abdominal infection, or drug induced following the administration of piperacillin/tazobactam. Additional considerations were made to a possible reaction following the mRNA COVID‐19 vaccine given the timing of the vaccine. Given the laboratory findings, a hemolytic cause was deemed unlikely due to the lack of schistocytes and elevated haptoglobin. Additionally, thrombotic thrombocytopenia purpura/hemolytic uremic syndrome was unlikely given the lack of schistocytes, renal dysfunction, or central nervous system dysfunction. The coagulation studies were not indicative of disseminated intravascular coagulation. A consumptive process following piperacillin/tazobactam use was deemed unlikely as the patient had no reported prior exposure to the antibiotic. Further, a reaction the mRNA COVID‐19 vaccine was deemed unlikely though could not be completely excluded. Lastly, given the unknown chronicity of his initial anemia and macrocytosis, neoplastic and bone marrow infiltrative processes were under consideration.

Months later it was discovered on report from the patient's wife that he had a bone marrow biopsy performed which had shown myelodysplastic syndrome. He had undergone treatment at an outside healthcare facility. While undergoing treatment for his MDS, the patient developed shortness of breath and found to have pneumonia. Unfortunately, he had passed away from complications of his pneumonia.

## 3 DISCUSSION

Pancytopenia is a hematologic condition characterized by a decrease in all three peripheral blood cell lines with a hemoglobin less than 11.5 g/dl in women and 13.5 g/dl in men, platelets of less than 150,000 per mcL, and leukocytes of less than 4000 per ml (or absolute neutrophil count of less than 1500–1800 per ml).^1^, ^2^ The myriad of causes for pancytopenia can be grouped into three major categories: (1) impaired production, (2) peripheral destruction, and (3) impaired production and peripheral destruction.^3^, ^4^ Within the causes of impaired production are aplastic anemia (acquired or congenital), bone marrow infiltrating disorders, chemotherapeutic drugs & radiation, and infection and nutritional deficiencies.^4^ The etiologies of peripheral destruction include autoimmune‐mediated pancytopenia which can have several inciting factors including medications and myelofibrosis and splenic sequestration.^4^, ^5^ Included in causes of combined impaired production and peripheral destruction is MDS, as in this patient's case as well as paroxysmal nocturnal hemoglobinuria, hemophagocytic lymphohistiocytosis, systemic lupus erythematosus, medications, and leukemia.^4^

In this patient's case, he was found to have myelodysplastic syndrome following further evaluation as an outpatient. Myelodysplastic syndrome is a clonal disorder of myeloid stem cells, which may occur de novo or secondary to various insults to the bone marrow.^6^ It typically arises from distorted hematopoietic stem cell function, inflammatory and innate immune deregulation, and multiple genomic events.^7^, ^8^ Several environmental and iatrogenic etiologies have been implicated, including exposure to chemotherapy, radiation, or environmental toxins such as benzene.^6^Chemotherapeutic agents such as alkylators or topoisomerase II inhibitors have been implicated as known causes of myelodysplastic syndromes, usually occurring 2 to 7 years after exposure.^9^ Clinical presentations of MDS are variable and include complications due to a variety of infections. Bacterial infections predominate with the skin being though most likely source, but fungal and viral causes may be seen as well.^10^ In this patient's case though he had an initial fever following his vaccine, he had shown no other definitive signs/proof of infection. Additionally, abnormalities of adaptive immunity may also occur even though lymphocytes are not generally derived from the malignant clone.^11^ In this patient's case, he was given a COVID mRNA vaccine approximately one week prior to hospital admission. His presentation questions whether his vaccine precipitated a maladaptive immune response ultimately unmasking his case of myelodysplastic syndrome. Currently, no cases of myelodysplastic syndrome following administration of an immunization have been identified in the literature. The actual preceding factor(s) for de novo myelodysplastic syndrome is not entirely understood but are assumed to occur from an oncogenic process resulting in one or more somatic mutations.^6^ More than 100 genes have been found to be recurrently mutated in myelodysplastic syndrome, and these encode spliceosome components, chromatin remodeling factors, epigenetic pattern modulators, and transcription factors among others.^12^ The most common somatic alterations include mutations in *TET2*, *SF3B1*, *ASXL1*, *DNMT3A*, *SRSF2*, *RUNX1*, *TP53*, *U2AF1*, *EZH2*, *ZRSR2*,*STAG2*, *CBL*, *NRAS*, *JAK2*, *SETBP1*, *IDH1*, *IDH2*, and *ETV6*.^13^ *TP53* mutations are associated with complex cytogenetics and poor overall survival. *RUNX1* and *TP53* tend to correlate with worse thrombocytopenia. *TET2* mutations have a better response to hypomethylating agents.^13^

Guidelines for the work‐up and evaluation for myelodysplastic syndrome include 2 pre‐requisite criteria for the diagnosis: (1) stable cytopenia for 6 months or longer, or 2 months if a certain karyotype or bilineage dysplasia is apparent, and (2) exclusion of other causes of dysplasia and/or cytopenia(s).^13^ Anemia is the most common manifestation, and this may be normocytic or macrocytic.^6^ Evaluation for other causes of anemia should be performed with additional laboratory testing including iron and ferritin levels, B12 and folate levels, hemolysis work‐up with lactate dehydrogenase (LDH), and haptoglobin.^14^ If clinically appropriate, it is also appropriate to perform Coombs testing, and serum protein electrophoresis (SPEP) and immunofixation (IFE).^14^ Histopathologic evaluation of the peripheral blood and bone marrow with a bone marrow aspirate and biopsy with the following criteria are required for diagnosis:

- One or more peripheral blood cytopenias (anemia, neutropenia, and/or thrombocytopenia) that cannot be explained by other causes, defined as hemoglobin less than 10 g/dL(100 g/L); absolute neutrophil count less than 1.8 × 10/L (less than 1800/microL); platelets less than 100 x 10/L (less than 100,000/microL).
- Blasts, which account for less than 20% of nucleated cells in the bone marrow and/or peripheral blood. If there is more than 20% blasts in the peripheral blood or bone marrow, myeloid sarcoma or presence of certain genetic findings including t(8;21), inv(16), or t(15;17), this is considered to be acute myeloid leukemia regardless of blast percentage.
- Evidence for dysplasia in greater than 10% of cell lines (red cell precursors, granulocytes, or megakaryocytes.^6^

Following histopathologic evaluation, a diagnostic evaluation should also include flow cytometry, immunophenotyping, evaluation of cytogenetics by karyotype and FISH, along with genetic profiling (performed with genomic profiling) to assess for relevant somatic mutations such as *SF3B1*, *TET2*, *SRSF2*, *ASXL1*, *DNMT3A*, *RUNX1*, *U2AF1*, *TP53*, and *EZH2*.^15^ Myeloblasts, which account for less than 20% of nucleated cells in the bone marrow and/or peripheral blood.

The prognosis of patients with myelodysplastic syndrome varies widely depending upon several characteristics including cytogenetics and severity of cytopenias.^13^ Patients with 5q‐ generally have a much better prognosis compared to monosomy 7, for example.^13^ The International Prognostic Scoring System (IPSS) and revised IPSS (R‐IPSS) are risk stratification system used by clinicians to guide treatment and the potential clinical course. These systems can be used in addition to a clinical assessment to determine the best therapeutic options. The IPSS includes the percentage of blasts in the bone marrow, karyotype, and the number of cell lineages with cytopenias.^13^ Karyotype with a good prognosis includes normal karyotype, ‐Y, deletion 5q, and deletion 20q.^13^ Poor risk karyotypes include complex cytogenetics (greater than three abnormalities) or chromosome 7 abnormalities.^13^ All other karyotypes are categorized as intermediate risk (Greenberg). Based on these findings, a score is calculated to determine a risk score of either low, intermediate‐1 or intermediate‐2, or high risk.^13^

Unique to this patient's differential diagnosis was the consideration of piperacillin/tazobactam‐induced pancytopenia, which is usually reversible. This toxicity is suspected to be dose‐related as the overwhelming majority of cases occurred following more than two continuous weeks of use.^16^ Previous data have indicated an inverse correlation between cumulative doses of piperacillin/tazobactam and the absolute neutrophil count.^16^ The suspected pathophysiologic cause of this is the arrest of myeloid cell development in response to piperacillin.^17^ Additionally, multiple cases of immune‐mediated thrombocytopenia and hemolytic anemia have been reported, which is suspected to be an immunoglobulin G (IgG) response to penicillin.^18^ In this patient's case, there was concern he may have developed an immune‐mediated pancytopenia in response to recent administration of piperacillin/tazobactam. Though this would not explain why the patient's leukopenia developed or why the patient did not have a hemolytic anemia.

As another diagnostic consideration though unlikely, the mRNA COVID‐19 vaccine was postulated to be a possible source of this patient's pancytopenia. Prior vaccines, including the hepatitis B vaccine, have had reported cases of pancytopenia following routine immunization. The proposed mechanism of action in this case is a dysregulation of CD8 T‐cells via increased interferon‐gamma from monocytes.^19^, ^20^ Cases of aplastic anemia have been reported in individuals who developed COVID‐19,^21^ but cases of pancytopenia have been reported in individuals who have received any of the available COVID‐19 vaccines including an mRNA vaccine. Currently, little to no information is known regarding the possible effects of the vaccine.^22^

## 4 CONCLUSION

Myelodysplastic syndrome is a clonal disorder of myeloid stem cells, which may occur de novo or secondary to various insults to the bone marrow (Arber). Several environmental and iatrogenic etiologies have been implicated, including exposure to chemotherapy, radiation or environmental toxins such as benzene (Arber). The actual preceding factor(s) for de novo myelodysplastic syndrome is not entirely understood. More than 100 genes have been found to be recurrently mutated in myelodysplastic syndrome, and these encode spliceosome components, chromatin remodeling factors, epigenetic pattern modulators, and transcription factors among others. International Prognostic Scoring System (IPSS) and revised IPSS (R‐IPSS) are risk stratification system used by clinicians to guide treatment and the potential clinical course for myelodysplastic syndromes.

## ACKNOWLEDGEMENT

This research was supported (in whole or in part) by HCA Healthcare and/or an HCA Healthcare affiliated entity. The views expressed in this publication represent those of the author(s) and do not necessarily represent the official views of HCA Healthcare or any of its affiliated entities.

## CONFLICT OF INTEREST

The authors have no conflicts to declare.

## AUTHORS CONTRIBUTIONS

Ian Lancaster performed literature review, reviewed case, and wrote the manuscript. Deep Patel wrote the manuscript and reviewed case. Vikas Sethi wrote the manuscript and reviewed case. Joseph Namey peer‐reviewed and assisted in writing manuscript. Weston Connelly peer‐reviewed and assisted in writing manuscript.

## DISCLAIMER

This research was supported (in whole or in part) by HCA Healthcare and/or an HCA Healthcare affiliated entity. The views expressed in this publication represent those of the author(s) and do not necessarily represent the official views of HCA Healthcare or any of its affiliated entities.

## CONSENT

Written informed consent was obtained from the patient to publish this report in accordance with the journal's patient consent policy.

## Data Availability

No data were used.
